# Development and validation of a postoperative delirium risk prediction model for non-cardiac surgery in elderly patients

**DOI:** 10.3389/fpsyt.2025.1414273

**Published:** 2025-04-28

**Authors:** Xu Lin, Na Tian, Yuanlong Wang, Shuhui Hua, Jian Kong, Shanling Xu, Yanan Lin, Chuan Li, Bin Wang, Yanlin Bi

**Affiliations:** ^1^ Department of Anesthesiology, Qingdao Municipal Hospital, Qingdao, China; ^2^ Department of Anesthesiology, The Eighth People’s Hospital of Qingdao, Qingdao, China; ^3^ The Second School of Clinical Medicine of Binzhou Medical College, Yantai, China; ^4^ Department of Anesthesiology, Weifang Medical College, Weifang, China

**Keywords:** non-cardiac surgery, elderly, delirium, risk factors, prediction model

## Abstract

**Background:**

Postoperative delirium (POD) is one of the common central nervous system complications in elderly patients after non-cardiac surgery. Therefore, it is necessary to develop and validate a preoperative model for POD risk prediction.

**Methods:**

This study selected 663 elderly patients undergoing non-cardiac elective surgery under general anesthesia for tracheal intubation in general surgery, from September 1^st^, 2020 to June 1^st^, 2022. Simple random sampling method was used according to 7: 3. The occurrence of POD within 1 to 7 days after the operation (or before discharge) was followed up by the confusion assessment method (CAM). This study innovatively included the pittsburgh sleep quality index (PSQI) and the numerical pain score (NRS) for clinical work, to explore the relationship between sleep quality and postoperative pain and POD. Univariate and Multivariable Logistic regression analysis was used to analyze stepwise regression to screen independent risk factors for POD. The creation of prediction models involved the integration of outcomes through the implementation of logistic regression analysis. In addition, internal validation is employed to ensure the reproducibility of the model.

**Results:**

A total of 663 elderly patients were enrolled in this study, and 131 (19.76%) patients developed POD. The incidence of POD in each department was not statistically significant. The predictors in the POD column line graph included age, Mini Mental State Examination (MMSE) score, history of diabetes, years of education, sleep quality index, ASA classification, duration of anesthesia and NRS score. The formula Z= 8.293 + 0.102 × age - 1.214 × MMSE + 1.285 × diabetesHistory - 0.304 × yearsOfEducation + 0.602 × PSQI + 1.893 × ASA + 0.027 × anesthesiaTime + 1.297 × NRS. Conducive to the validation group to evaluate the prediction model, the validation group AUC is 0.939 (95% CI 0.894-0.969), the sensitivity is 94.44%, and the specificity is 85.09%. The calibration curves show a good fit between the clinically predicted situation and the actual situation.

**Conclusion:**

The clinical prediction model constructed based on these independent risk factors has a good predictive performance, which can provide reference for the early screening and prevention of POD in clinical work.

**Trial registration:**

ChiCTR2000033639 Retrospectively registered (date of registration: 06/07/2020).

## Introduction

Postoperative delirium (POD) is one of the common central nervous system complications after surgery. It is defined as an acute encephalopathy syndrome, which is manifested by sharp fluctuations in mental state, and its onset is rapid (a few hours to a few days) and the condition fluctuates rapidly (1). Common symptoms include: confusion, decreased concentration, disorientation, cognitive decline, changes in personality and mood, and even delusions and hallucinations (2). POD usually occurs 1-7 days after surgery, of which the incidence is higher after 1-3 days (3). The occurrence of POD significantly prolongs the patient’s hospital stay and the readmission rate, which increases the economic burden of patients and also leads to an increase in postoperative mortality (4). At present, the pathogenesis of POD is not clear. The following pathogenesis theories are generally accepted by researchers: cholinergic theory, inflammatory response theory, stress response theory, abnormal energy metabolism, exogenous toxins etc. (5) But more and more studies have shown that the occurrence of POD is largely affected by patient-related risk factors, which accumulate in different age groups and regions (6).

In recent years, more and more researchers have conducted research on POD to explore the development process of POD, which can be effective prevention and treatment measures for POD. So far, the identified risk factors that affect the occurrence of POD include age, education level, underlying disease, pain and sleep quality (7–11). Advanced age is considered to be an independent risk factor for patients with POD, and the incidence of POD increases significantly with age (7). Education level and baseline level of preoperative cognition are related to POD. Patients with poor baseline knowledge before surgery have a higher incidence of POD (8, 12). Diabetes and preoperative hyperglycemia may be related to the increased risk of POD, which may be related to neuropathy induced by hyperglycemia (9). Postoperative pain may cause mental stress and sleep disturbance, increasing the risk of POD. Properly controlling pain after surgery can reduce the risk of POD (10, 13). Disturbance of the postoperative sleep cycle and changes in the patient’s living environment may cause postoperative sleep disorders in some patients, and may induce or aggravate POD (11).

Risk factors such as advanced age, education level, pain, and sleep quality affect the occurrence and development of POD. Therefore, it is necessary to determine the risk factors involved in POD, so as to provide clinicians with the ability to implement nursing plans and preventive measures (14). Clinical prediction models (CPMs) mainly refer to the use of mathematical formulas to estimate the probability of an individual currently suffering from a certain disease or a certain outcome in the future. The clinical prediction model can predict the possibility of a certain disease to a certain extent, in order to assess the risk of disease and take preventive measures in time.

So far, research shows that some clinical prediction models have been developed clinically, but these clinical prediction models have limitations in terms of repeatability and extrapolation (15, 16). So the POD prediction model with good repeatability and extrapolation is very important.

For the above reasons, a cohort study was performed to analyze the related risk factors of POD, establish a POD clinical prediction model with good repeatability and extrapolation, which could predict and evaluate the risk of POD.

## Materials and methods

### Study design

This study is a prospective cohort study. It was performed in accordance with the Declaration of Helsinki and was approved by the Ethics Committee of Qingdao Municipal Hospital, which was registered on ClinicalTrials.gov (ChiCTR2000033639). Then written informed consents from all subjects or legal surrogates were obtained.

This study selected 663 elderly patients undergoing non-cardiac elective surgery under general anesthesia for tracheal intubation in general surgery, orthopedics, urology, hepatobiliary and pancreatic surgery in our hospital from September 1st, 2020 to June 1st, 2022. Simple random sampling method was used according to 7: 3. The proportions divided the patients into the development group 464 cases and the validation group 199 cases. The clinical data of the patients before, during and after the operation were collected, and the occurrence of POD within 1 to 7 days after the operation (or before discharge) was followed up.

### Study inclusion and exclusion criteria

The study was conducted in Qingdao Municipal Hospital (Qingdao, China) between September 1^st^, 2020 and June 1^st^, 2022 We selected eligible elderly patients who from General Surgery, Orthopedics, Urology, Hepatobiliary and Pancreatic Surgery, were 65 to 90 years old, and were selected for elective surgery under general anesthesia for tracheal intubation, ASA grade I~ III, good preoperative cognitive status and no language communication barriers, and also able to complete preoperative cognitive function tests. Patients with any of the following conditions are not included in this study: (1) Patients undergoing major operations such as emergency surgery or cardiovascular surgery within one month; (2) Central nervous system infections, head trauma, epilepsy, multiple sclerosis and other major operations Nervous system diseases;(3) Patients with uncontrolled cardiovascular (New York Heart Association classification of heart grade 3 or 4) and cerebrovascular diseases (mini-mental state examination (MMSE) <24 points), severe liver (aspartate transaminase and alanine transaminase > 40) and kidney dysfunction (Creatinine > 120, urea nitrogen > 7.2), and hemorrhagic diseases (Prothrombin time > 14, activated partial coagulation > 37); (4) Patients with vital organ failure, mental or consciousness disorders; (5) Long-term use of psychotropic drugs, Steroid drugs, hormone drugs; (6) Preoperative MMSE <24 points;(7) The patient was transferred to the intensive care unit (ICU) after the operation. Research exclusion criteria: (1) the patient voluntarily withdrew from the study; (2) the patient was unable to cooperate with the postoperative follow-up work; (3) the postoperative loss to follow-up.

### Anesthesia and surgery

The cohort study involved patients who had not taken any medication before the surgery and had fasted for 8 hours and abstained from water for 6 hours. Vital signs, including electrocardiogram, pulse oximetry, and noninvasive blood pressure, were routinely monitored prior to anesthesia administration. Peripheral venous access was established. Venous blood samples were collected from 6am to 7am on the day of surgery.

Patients received general anesthesia via tracheal intubation. The anesthetic induction agents administered were as follows: sufentanil at a dose of 0.2 ~ 0.5 μg/kg, cis-atracurium at a dose of 0.15 ~ 0.2 mg/kg, etomidate at a dose of 0.15 ~ 0.3 mg/kg, and dexmedetomidine, which was continuously infused at a dose of 0.2 ~ 0.5 μg/kg/hr during the surgical procedure. The infusion was stopped 30 minutes prior to the end of surgery. Analgesia was maintained through continuous infusion of remifentanil at a rate of 0.252 μg/kg/min. Intermittent cisatracurium was added at 40-minute intervals after induction and discontinued 1 hour prior to the conclusion of surgery. Administration of sevoflurane via inhalation anesthesia ranged from 0.5% to 3% depending on the depth of anesthesia. After the surgical procedure, patients were transferred to the post-anesthetic care unit (PACU) following the extubation process. They remained in the PACU for 30 minutes to monitor their recovery before being moved back to the ward.

### Perioperative risk factors and diagnosis of delirium

#### Perioperative risk factors

Combined with the risk factors found in clinical work and related studies (1–6), this study included 33 risk factors for analysis. The risk factors are listed as follows: Gender, age, MMSE, underlying disease (hypertension, diabetes, coronary heart disease), smoking history, drinking history, years of education, pittsburgh sleep quality index (PSQI), department, preoperative hemoglobin level, preoperative total Protein level, preoperative albumin level, preoperative blood glucose level, preoperative blood sodium concentration, preoperative blood potassium concentration, preoperative blood calcium concentration, BMI), ASA classification, sufentanil dosage, sevoflurane alkane dosage, fluid transfusion, blood transfusion history, red blood cell transfusion, plasma transfusion, blood loss, urine output, intraoperative average body temperature, intraoperative hypotension, operation time, anesthesia time, numerical pain score (NRS).

### Diagnosis of delirium

Researchers who have undergone professional scale evaluation and training will use the confusion assessment method (CAM) (18) on the 1-7 days after the operation (or before discharge). Patients who were later transferred to the ward were followed up twice a day. The follow-up period was 9:00~10:00 in the morning and 14:00~15:00 in the afternoon. The CAM is the international standard for diagnosis of POD, including the following delirium characteristics: (1) acute onset with a fluctuating course; (2) inattention; (3) disordered thinking, and (4) changes in the level of consciousness (any state of consciousness other than fully conscious). The diagnosis of delirium requires the presence of (1) and (2), accompanied by (3) or (4) or both. Patients with at least once POD positive 1 to 7 days after operation (or before discharge) were included in the POD group, and patients with no POD 1 to 7 days after operation (or before discharge) were included in the non-POD group.

### Neuropsychological tests and other related scales

MMSE was performed the day before the operation. The scale includes the following 7 aspects: time orientation, place orientation, immediate memory, attention and calculation, delayed memory, language, and visual space. It can comprehensively, accurately and quickly reflect the mental state of subjects and the degree of cognitive impairment. Therefore, it is widely used as a screening tool for dementia and a measure of overall cognitive function.

PSQI: PSQI score was also performed the day before the operation. And it is used to assess the sleep quality of subjects in the past month. 19 self-assessed and 5 other-rated items constitute 7 parts such as sleep quality and sleep efficiency. Each part is scored from 0 to 3, and the total score ranges from 0 to 21. The higher the score, the worse the sleep quality.

NRS: Using numbers 0-10 instead of words to indicate the degree of pain. 0 points for no pain, 1 to 3 points for mild pain, 4 to 6 points for moderate pain, 7 to 9 points for severe pain, and 10 points for severe pain. Postoperative NRS scores were visited by researchers trained in the evaluation of the NRS scale at 24 hours postoperatively and the NRS scores were recorded. If the NRS score of patients ≥3 points during the postoperative follow-up period, the patient will be given non-steroidal analgesics to relieve the pain.

### Statistical analysis

According to the pre-experimental analysis, it is estimated that there are 8 independent risk factors affecting the occurrence of POD, and we need 10 cases of delirium in the training set for every predictor they include in the model and want to put 8 predictors in the model, with using 70% of enrolled patients in the training set, assuming a 21% incidence and a 20% loss-to-follow-up rate and supposing 0.8 of the power. Therefore, the calculated sample size is 686 cases (8×10÷21%×120%÷70%). According to the exclusion and exclusion criteria, 24 patients were excluded from this study. According to the 7:3 principle (17), the sample size is divided into development group and validation group. In this study, the development group and validation group included 464 cases and 199 cases respectively.

Combining the confirmed risk factors of clinical and related studies as independent variables to reduce the influence of confounding factors on the subsequent regression analysis, the independent variables were subjected to univariate logistic regression analysis, and the variables with *P*<0.1 were established to be included in the subsequent Logistic regression model; Multivariable logistic regression analysis was used stepwise regression analysis further screens independent risk factors, and establishes the final Logistic regression model according to *P*<0.05. The stepwise regression in this study used AIC information statistic as a criterion to obtain the optimal model by selecting the lowest AIC. carries out risk assignment according to the β value, calculates the POD risk score, and obtains the POD risk prediction formula; Using R4.4.3 software to draw nomogram (Nomogram plot), calibration curve (Calibration Curve). In the calibration curves, Apparent curves were obtained by training and Bias-corrected curves were obtained by training after repeated autonomous sampling of samples. The prediction model established by the development group is used to draw the calibration curve, and the area under the receiver operating characteristic curve (AUC) of the prediction model of the validation group and the sensitivity and specificity under the optimal threshold are calculated to evaluate the prediction effect of the model.

## Results

**Figure 1:**
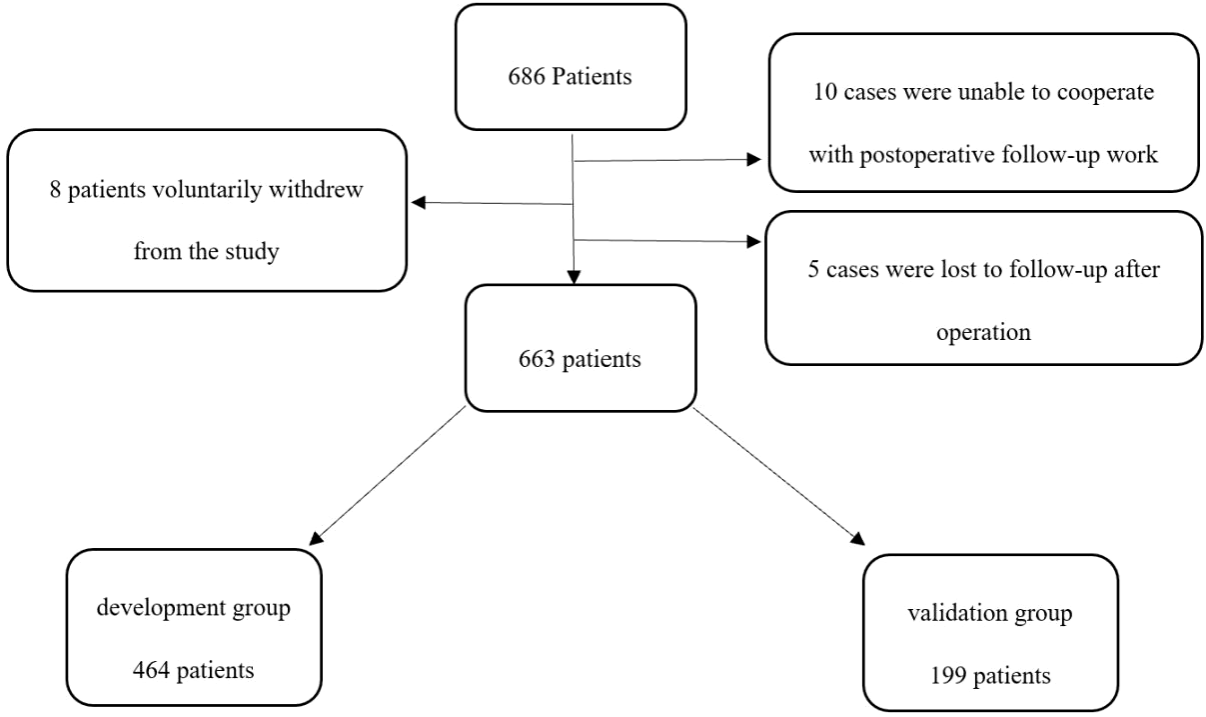
Flow diagram. The flow diagram shows that 686 patients were initially screened for the studies, and 663 patients were finally included in the data analysis.

In this study, the number of subjects who signed the informed consent form was 686, 8 patients voluntarily withdrew from the study, 10 cases were unable to cooperate with postoperative follow-up work, 5 cases were lost to follow-up after operation, and 663 patients were finally included in this study. (**Figure 1**) According to the CAM method, 131 cases were diagnosed as positive for POD, and the incidence of POD was 19.76%.

Compared with the non-POD group in the development group, the POD group has statistical differences in age, MMSE, history of hypertension, diabetes, history of coronary heart disease, history of drinking, years of education, PSQI, preoperative hemoglobin level, Preoperative total protein level, preoperative albumin level, preoperative blood glucose level, preoperative blood sodium concentration, BMI, ASA classification, fluid transfusion, blood transfusion history, red blood cell transfusion, plasma transfusion, blood loss, Urine volume, average body temperature during operation, whether hypotension occurred during operation, operation time, anesthesia time, NRS score (*P*<0.1) (**Table 1**).

**Table 1:** Univariate logistic regression analysis of POD group and non-POD group in developmental group.

Variable	POD Group(n=90)	Non-POD Group(n=374)	OR Value(CI 95%)	*P*
Male ratio(case, %)	64.67	59.27		0.107
Age(year)	76 ± 6	71 ± 5	1.146(1.023~1.289)	<0.001
MMSE (score)	24 ± 1	27 ± 1	0.220(0.090~0.538)	<0.001
Hypertension(case, %)	34(37.77)	160(42.78)	1.318(1.065~1.564)	<0.001
Diabetes(case, %)	42(46.67)	73(19.51)	1.806(1.023~1.889)	<0.001
CHD(case, %)	42(46.67)	84(22.45)	1.505(1.088~1.914)	<0.001
Smoking(case, %)	33(36.67)	143(38.23)	1.524(1.308~1.550)	0.215
Drinking(case, %)	38(42.22)	107(28.61)	1.442(1.248~1.942)	0.073
Years of education(year)	6 ± 2	10 ± 3	0.691(0.497~0.959)	<0.001
PSQI (score)	12 ± 3	7 ± 2	2.021(1.431~2.854)	<0.001
Department(case, %)				0.762
General Surgery	30(33.33)	115(30.75)	1.436(1.123~1.759)	
Orthopedics	23(25.56)	90(24.06)	1.467(1.054~1.858)	
Urology	25(27.78)	109(29.14)	1.768(1.133~1.955)	
Hepatobiliary Surgery	12(13.33)	60(16.04)	1.537(1.169~1.842)	
Hemoglobin(g/L)	118.89 ± 21.89	131.92 ± 17.96	1.019(1.002~1.074)	<0.001
Total protein(g/L)	62.62 ± 5.80	65.47 ± 4.95	0.925(0.756~0.989)	<0.001
Albumin(g/L)	36.58 ± 3.80	38.74 ± 3.16	0.988(0.723~0.995)	<0.001
Blood sugar(mmol/L)	6.26 ± 2.28	5.68 ± 1.77	1.870(1.523~1.923)	0.012
Blood sodium(mmol/L)	139.45 ± 2.90	140.14 ± 2.37	0.980(0.769~0.994)	0.024
Serum potassium(mmol/L)	3.97 ± 0.33	3.97 ± 0.37	0.487(0.373~0.856)	0.847
Blood calcium(mmol/L)	2.24 ± 0.13	2.26 ± 0.11	2.117(1.021~2.865)	0.172
BMI(Kg/m^2^)	23.78 ± 3.84	24.94 ± 3.51	0.812(0.685~0.962)	0.007
ASA Grade(case, %)				<0.001
I	0(0.00)	23(6.14)	0.012(0.002~0.128)	
II	46(51.11)	321(85.83)	1.143(1. 045~1.573)	
III	44(48.89)	31(8.29)	1.857(1.648~2.053)	
Sufentanil dosage(μg)	21 ± 2	19 ± 3	0.880(0.619~0.982)	0.735
Sevoflurane dosage(ml)	58 ± 8	62 ± 6	1.246(1.112~1.492)	0.018
Infusion volume(mL)	2289 ± 157	1819 ± 182	0.970(0.965~0.997)	<0.001
Blood transfusion(case, %)	17(18.89)	16(4.28)	1.640(1.370~2.453)	<0.001
Red blood cell transfusion(U)	0.81 ± 0.38	0.39 ± 0.08	1.978(1.242~2.359)	<0.001
Plasma transfusion(mL)	129 ± 36	63 ± 13	0.994(0.974~0.998)	<0.001
Bleeding volume(mL)	226 ± 43	136 ± 30	0.993(0.897~0.999)	<0.001
Urine volume(mL)	702 ± 80	464 ± 40	1.002(1.001~1.005)	<0.001
Intraoperative mean temperature(°C)	36.2 ± 0.3	36.3 ± 0.3	0.368(0.227~0.829)	0.002
Intraoperative hypotension(case, %)	58(64.44)	179(47.86)	1.240(1.051~1.545)	0.094
Operation time(min)	284 ± 14	176 ± 13	1.039(1.007~1.937)	<0.001
Anesthesia time(min)	306 ± 23	233 ± 32	1.082(1.078~1.990)	<0.001
NRS score	3 ± 1	1 ± 1	2.179(1.823~2.777)	<0.001

Values are expressed as number (%), mean ± standard deviation.

The length of anesthesia was defined from the time that the anesthesiologists started general anesthesia in the patients to the time when the patients were sent to the post-anesthesia care unit. The length of surgery was defined from the time of initial incision to the time of the closure of the skin.

ASA, American Society of Anesthesiologists; BMI, Body Mass Index; CHD, Coronary Heart Disease; MMSE, Mini-mental State Scale; NRS, Numeric Rating Scales; PSQI, Pittsburgh Sleep Quality Index.

The factors of *P*<0.1 in the univariate logistic regression analysis were included in the Multivariable logistic regression stepwise regression analysis. The results showed that age, MMSE, diabetes history, years of education, PSQI, ASA classification, surgical anesthesia time, NRS score for non-cardiac surgery independent risk factors for POD in elderly patients (*P*<0.05) (**Table 2**).

**Table 2:** Multivariable logistic regression stepwise regression analysis of the development group.

Independent risk factors	Regression coefficient	OR Value(CI 95%)	*P*
Age	0.102	1.121(1.005~1.209)	0.036
MMSE Score	-1.214	0.350(0.185~0.587)	<0.001
Diabetes	1.285	3.932(1.130~6.436)	0.042
Years of education	-0.304	0.824(0.592~0.937)	0.011
PSQI	0.602	1.846(1.393~2.301)	<0.001
ASA Grade	1.893	4.176(2.012~4.315)	0.002
Anesthesia time	0.027	1.032(1.006~1.018)	<0.001
NRS score	1.297	2.983(1.549~3.839)	0.001

The constant is 8.2934.

ASA, American Society of Anesthesiologists; MMSE, Mini-mental State Scale; NRS, Numeric Rating Scales; PSQI, Pittsburgh Sleep Quality Index.

**Figure 2:**
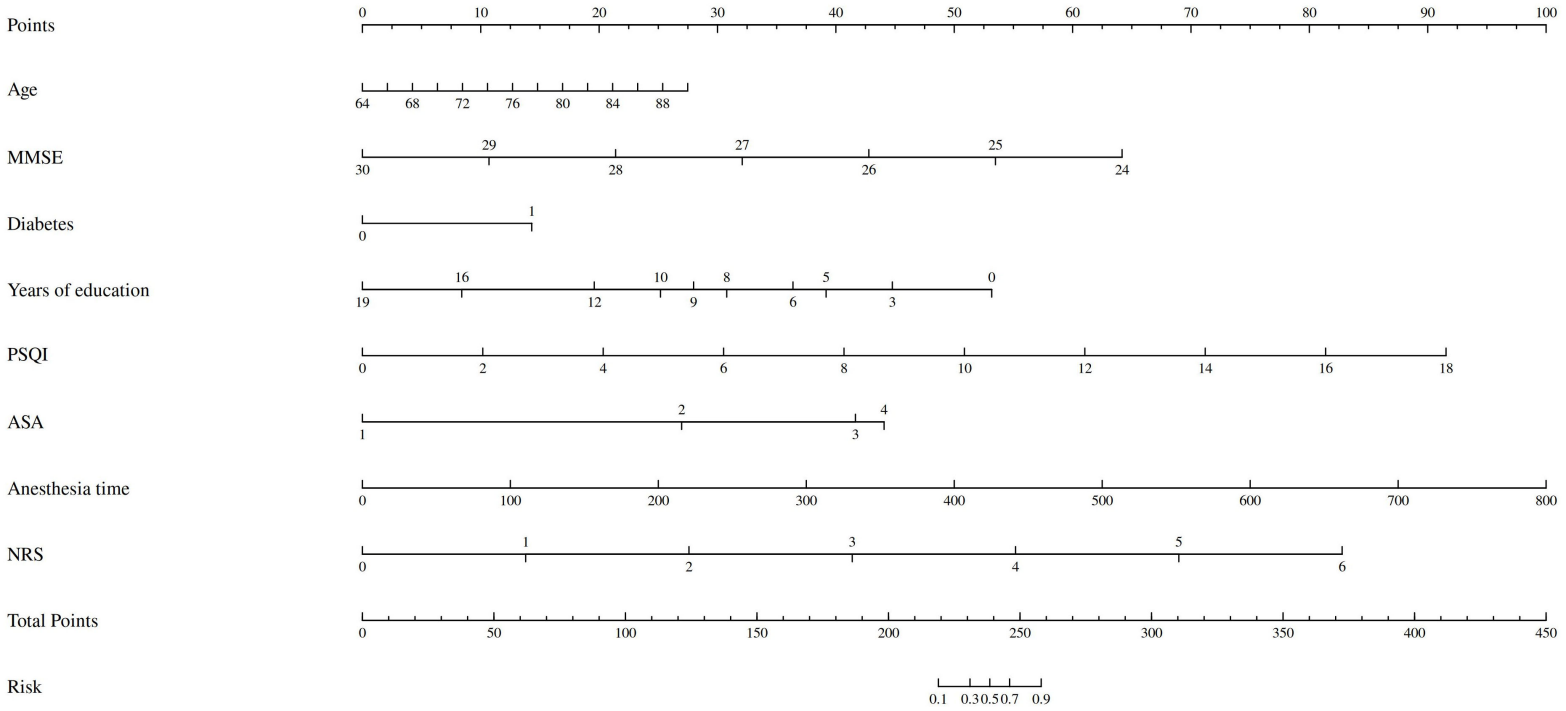
The nomogram of the non-cardiac surgery elderly prediction model.

At the same time, the nomogram is drawn through R 3.6.3 (**Figure 2**), and the total score is calculated by adding the corresponding scores of each item to obtain the probability of POD. According to the Multivariable Logistic regression stepwise regression analysis of the development group POD group, the predicted value of the development group is calculated. The formula Z= 8.293 + 0.102 × age - 1.214 × MMSE + 1.285 × diabetesHistory - 0.304 × yearsOfEducation + 0.602 × PSQI + 1.893 × ASA + 0.027 × anesthesiaTime + 1.297 × NRS.

**Figure 3:**
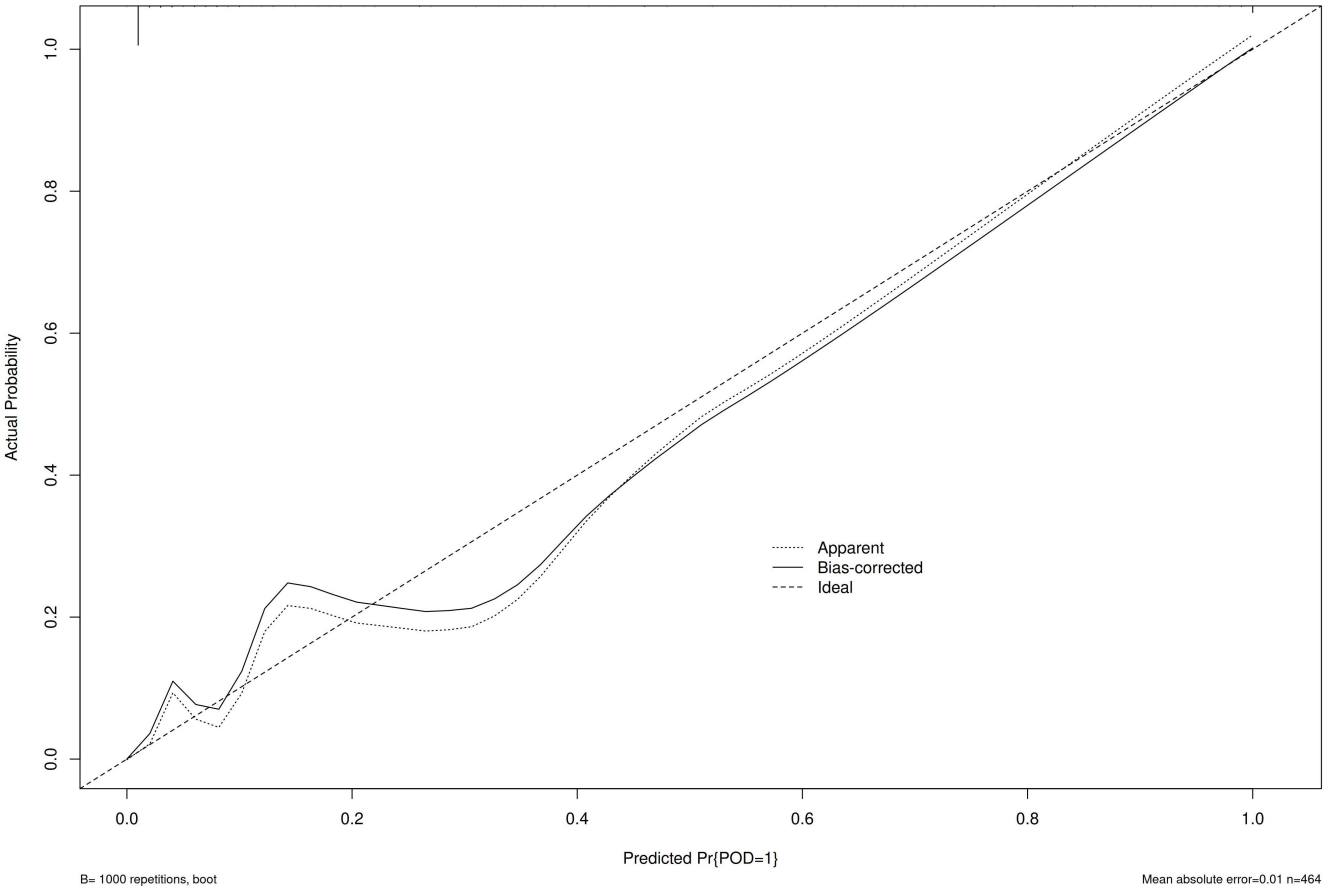
Calibration curve of the development group.

**Figure 4:**
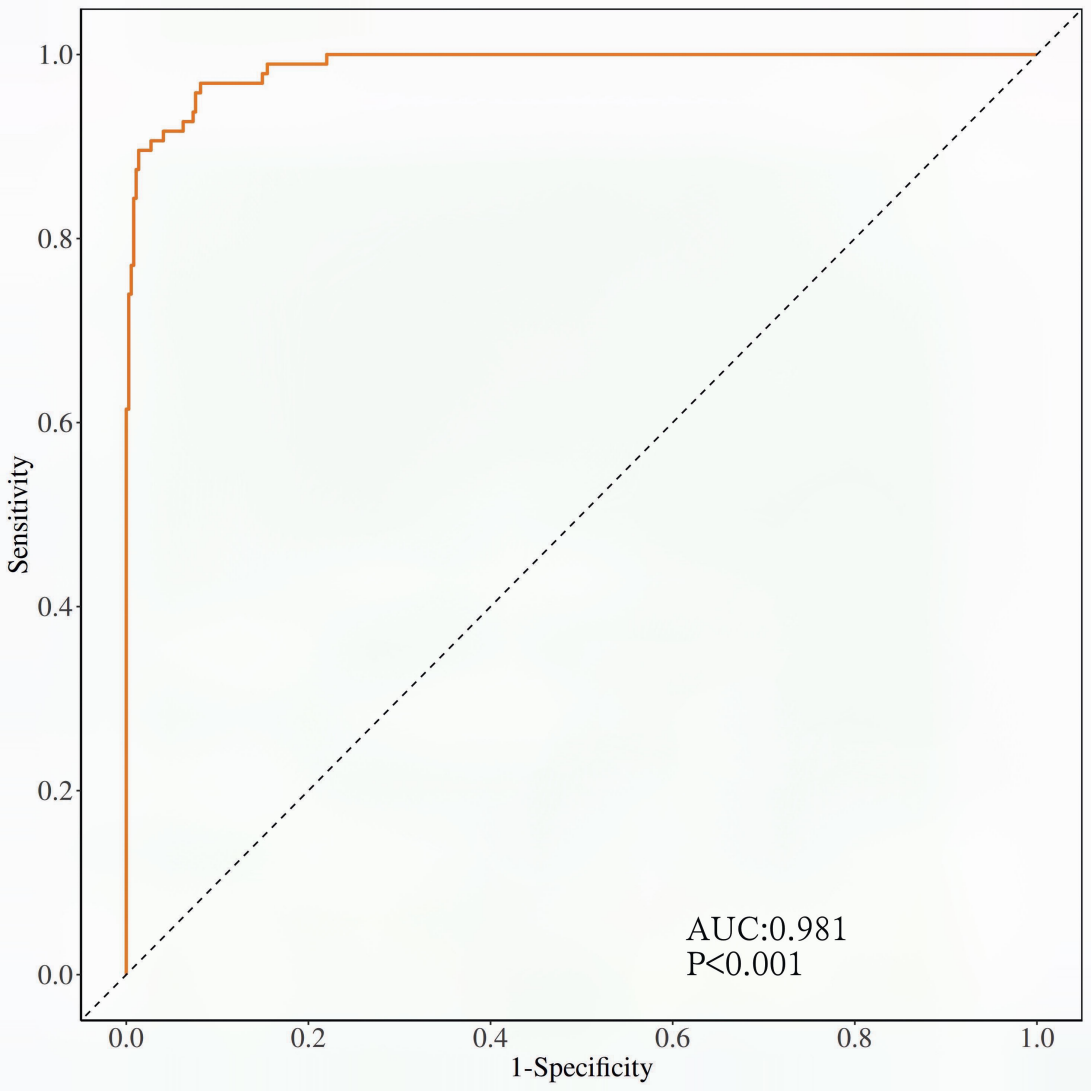
ROC curve of the development group forecasting model.

The calibration curve (**Figure 3**) and ROC curve (**Figure 4**) were drew according to the predicted values of the development group, and analyze the degree of fit and discrimination between the predicted situation and the actual situation. According to the results of the Calibration curve, the prediction of the development group has a high degree of fit with the actual situation. The ROC curve showed that the AUC was 0.981, the Youden index was 0.881, the sensitivity was 95.95%, and the specificity was 92.92%.

Compared with the validation group, the development group had statistical differences (*P*<0.05) except for the amount of fluid infusion, history of blood transfusion, and the amount of red blood cells transfused (*P*>0.05) (**Table 3**).

**Table 3:** Comparison of indicators between the development group and the validation group.

Variable	Development group (n=464)	Validation group (n=199)	*P*
Male ratio (case, %)	59.29	57.54	0.691
Age (year)	71 ± 7	70 ± 6	0.613
MMSE score	27 ± 1	26 ± 2	0.315
Hypertension (case, %)	222 (47.84)	90 (45.22)	0.536
Diabetes (case, %)	113 (24.35)	49 (24.62)	0.922
CHD (case, %)	122 (26.29)	45 (22.61)	0.334
Smoking (case, %)	173 (37.28)	83 (41.70)	0.291
Drinking (case, %)	147 (31.68)	67 (33.67)	0.644
Years of education (year)	9 ± 3	9 ± 2	0.987
PSQI	8 ± 2	8 ± 2	0.214
Department (case, %)			0.772
General Surgery	143 (30.82)	61 (30.65)	
Orthopedics	113 (24.35)	49 (24.62)	
Urology	136 (29.31)	58 (29.15)	
Hepatobiliary Surgery	72 (15.52)	31 (15.58)	
Hemoglobin (g/L)	129.13 ± 19.58	131.7 ± 21.25	0.145
Total protein (g/L)	64.86 ± 5.27	64.97 ± 5.94	0.825
Albumin (g/L)	38.28 ± 3.42	38.51 ± 3.69	0.456
Blood sugar (mmol/L)	5.80 ± 1.90	5.98 ± 2.22	0.330
Blood sodium (mmol/L)	139.99 ± 2.50	139.88 ± 2.48	0.609
Serum potassium (mmol/L)	3.97 ± 0.36	4.01 ± 0.36	0.253
Blood calcium (mmol/L)	2.26 ± 0.12	2.25 ± 0.11	0.571
BMI (Kg/m^2^)	24.69 ± 3.61	25.10 ± 3.68	0.209
ASA Grade (case, %)			0.351
I	23 (4.96)	9 (4.52)	
II	366 (78.88)	159 (79.90)	
III	75 (16.16)	31 (15.58)	
Sufentanil dosage (μg)	21 ± 2	19 ± 3	0.735
Sevoflurane dosage (ml)	58 ± 8	62 ± 6	0.018
Infusion volume (mL)	1254 ± 257	1519 ± 282	<0.001
Blood transfusion (case, %)	36 (7.76)	27 (13.57)	0.021
Red blood cell transfusion (U)	0.52 ± 0.14	0.79 ± 0.29	<0.001
Plasma transfusion (mL)	79 ± 20	97 ± 30	0.075
Bleeding volume (mL)	111 ± 34	120 ± 38	0.171
Urine volume (mL)	503 ± 46	543 ± 93	0.132
Intraoperative mean temperature (°C)	36.2 ± 0.3	36.2 ± 0.4	0.272
Intraoperative hypotension (case, %)	237 (51.08)	97 (48.74)	0.624
Operation time (min)	208 ± 18	244 ± 11	0.102
Anesthesia time (min)	278 ± 29	293 ± 24	0.236
NRS score	2 ± 1	2 ± 1	0.186

Values are expressed as number (%), mean ± standard deviation.

The length of anesthesia was defined from the time that the anesthesiologists started general anesthesia in the patients to the time when the patients were sent to the post-anesthesia care unit. The length of surgery was defined from the time of initial incision to the time of the closure of the skin.

ASA, American Society of Anesthesiologists; BMI, Body Mass Index; CHD, Coronary Heart Disease; MMSE, Mini-mental State Scale; NRS, Numeric Rating Scales; PSQI, Pittsburgh Sleep Quality Index.

**Figure 5:**
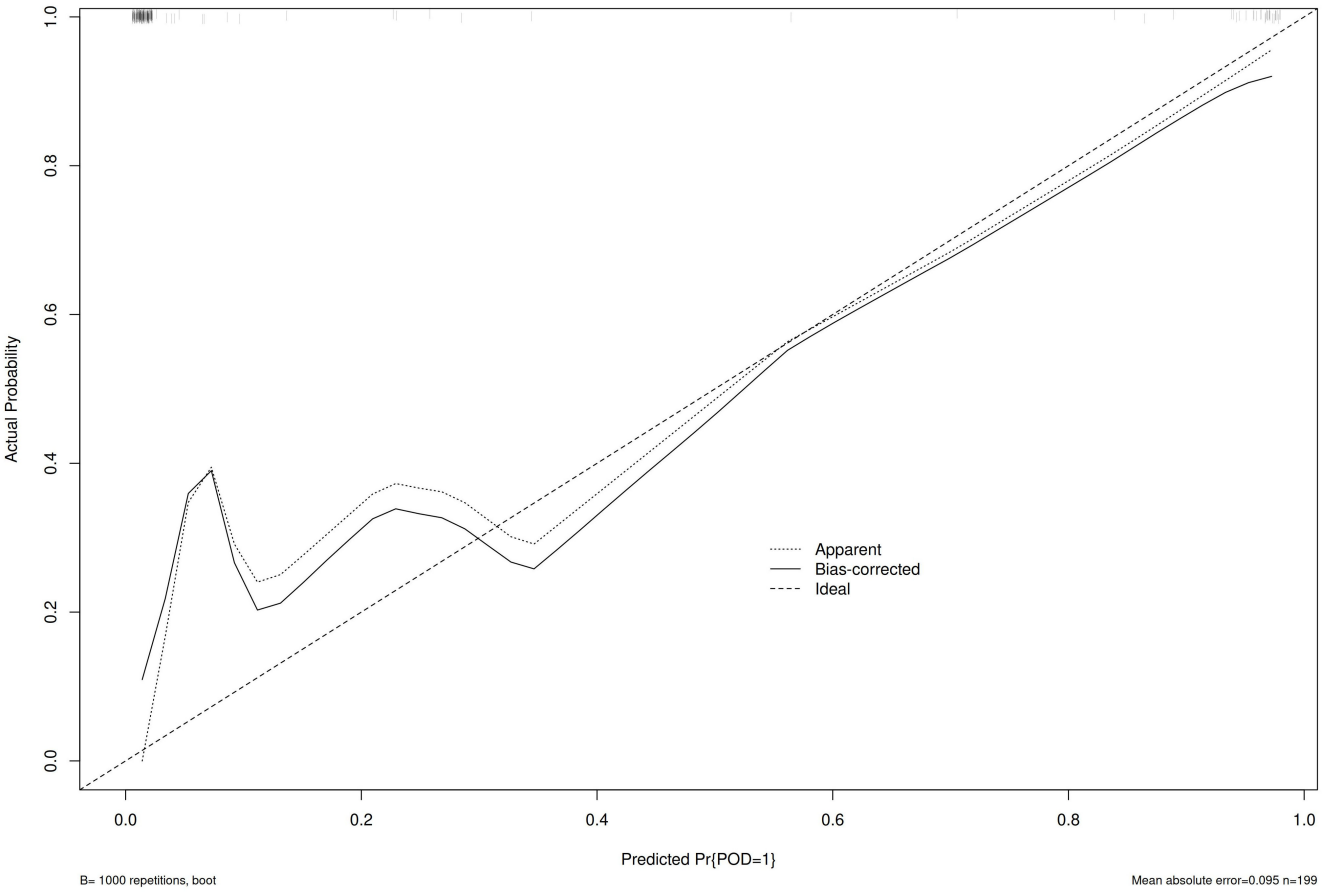
Validation group calibration curve.

**Figure 6:**
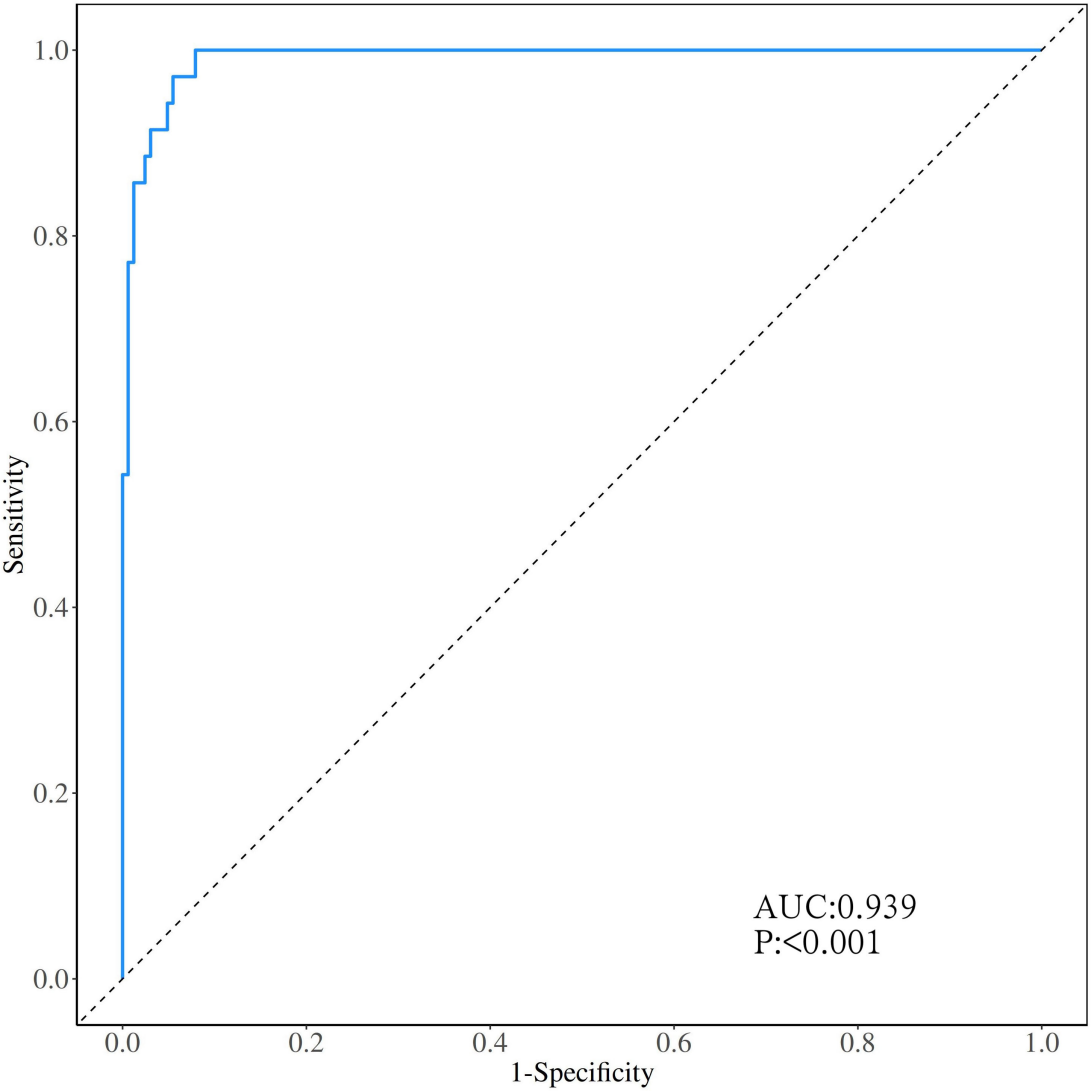
ROC curve of the validation group prediction model.

Bring the prediction formula obtained from the Multivariable Logistic regression stepwise regression analysis of the development group POD group into the validation group, predict the occurrence of POD in the validation group, draw the Calibration curve (**Figure 5**) and the ROC curve (**Figure 6**) according to the calibration curve results. The prediction situation of the verification set has a higher degree of fit with the actual situation. The ROC curve showed that the AUC was 0.939, the Youden index was 0.795, the sensitivity was 94.44%, and the specificity was 85.09%.

The magnitude of clinical utility in identifying patients with this predictive model over the use of screening using age and ASA score, which is calculated to be 0.4523 (95%CI: 0.4003-0.5044, *P*<0.01) using integrated discrimination improvement (IDI) score.

## Discussion

POD as one of the common postoperative complications, has attracted widespread attention because of its rapid onset and rapid fluctuations in cognitive function. POD can not only cause a decrease in patients’ attention, orientation and cognitive function, but also prolong the length of hospital stay, increase medical expenses, and increase patient mortality (19). The results of this study show that the incidence of POD is 19.76%, which is basically in line with the results of previous related studies (20).

POD is a complex disease caused by individual differences and multiple factors. In addition to individual differences, many risk factors affect the occurrence and development of POD, increasing the risk and incidence of POD. There are many risk factors that affect the occurrence and development of POD. Among them, advanced age is a risk factor confirmed by many studies (21, 22). This study also shows that advanced age is an independent risk factor for POD. This may be because advanced age leads to different degrees of atherosclerosis. Atypical sclerosis affects the blood supply of the brain, leading to degenerative changes in the central nervous system, making elderly patients more prone to POD; elevated blood glucose levels before surgery and history of diabetes are also independent risk factors for POD, which may be caused by neurodegeneration induced by high blood glucose levels (23, 24). The results of this study show that shorter years of education and low MMSE are independent risk factors for POD, which are consistent with previous research results (25, 26), that is, education level and baseline level of preoperative cognition are related to POD. The incidence of POD is higher in patients with education level and poor baseline level of preoperative cognition; a high ASA grade is also an independent risk factor for POD. A high ASA grade indicates that the patient has more underlying diseases, more severe organ dysfunction, and more critical illness. Thereby increasing the incidence of POD (27); previous studies have shown that longer surgical anesthesia time is a risk factor for POD (28), this study also suggests that surgical anesthesia time is an independent risk factor for POD, which may be related to longer surgical anesthesia time increases the body’s metabolic load. Long-term surgical stimulation and the interaction of a variety of drugs to maintain anesthesia may make patients more prone to POD. Previous studies have found that sleep cycle disturbances and changes in the living environment of patients may cause sleep disorders in some patients, and induce or aggravate POD (29); at the same time, postoperative pain is related to the occurrence and development of POD, and postoperative pain may cause mental stress and stress. Sleep disturbances increase the risk of POD, and reasonable control of pain after surgery can reduce the risk of POD (30). The innovation of this study is to incorporate commonly used scales for evaluating sleep quality and pain degree into the study to explore the correlation between sleep quality and postoperative pain and POD. In this study, the PSQI and pain digital score respectively reflected the patient’s sleep quality and postoperative pain, and also suggested that postoperative pain and sleep disturbance are independent risk factors for POD.

At present, there is no effective treatment for POD, and prevention is the main focus in clinical practice. Therefore, early screening of POD and prediction of the probability of POD have important clinical significance. The clinical prediction model can provide doctors with good clinical screening and preventive measures for the patient’s possible development of a certain disease. The establishment of a POD clinical prediction model can easily and efficiently predict and evaluate the probability of POD in clinical work. In this study, univariate and Multivariable Logistic regression stepwise regression analysis was used to establish a POD clinical prediction model, and at the same time, the R software was used to draw a nomogram to visualize the clinical prediction model. Each independent risk factor gets corresponding different scores on the top score line through the vertical line, and then the scores of all risk factors are added together to get the total score, which can correspond to the probability of POD for different individuals.

At the same time, this study draws the Calibration curve and ROC curve based on the predicted values of the development group and the validation group to evaluate and verify the fit and discrimination between the predicted situation and the actual situation of the established clinical prediction model. Different from other clinical prediction models (31, 32), this study uses the Calibration curve to verify the degree of fit between the prediction of the clinical prediction model and the actual situation. Compared with Calibration Plot, the Calibration curve visualizes the results of the goodness of fit test. The closer the prediction curve is to the ideal curve, it indicates that the prediction situation has a higher degree of fit with the actual situation, and it is more intuitive to observe the degree of fit between the predicted situation of the clinical prediction model and the actual situation; this study uses the ROC curve to verify the clinical model. The diagnostic value of the prediction model is judged by the AUC, Youden index, sensitivity, and specificity, which indicates that this prediction model with good repeatability and extrapolation distinguishes their work from prior work in this field (15, 16).

The clinical value of the model presented still requires further justification. The difference between statistical prediction and clinical utility is required. Therefore, the magnitude of clinical utility in identifying patients with the predictive model is better than the use of screening using age and ASA score, which is calculated by IDI score. So the predictive model could improve service delivery to people who require it. And the extra effort required to collect the MMSE and PSQI is justified. Meanwhile, the improvement in predictive performance worth the extra cost required to collect the MMSE and PSQI.

This prediction model explores the risk factors of POD which have been confirmed in clinical and related studies, and constructs a predictive model with higher predictive performance, which provides a reference for clinical work. This study innovatively included the MMSE, PSQI and NRS, three convenient scales for clinical work. Therefore, comparing their models that only used routinely collected variables (33–35), we suggest these variables be measured routinely in all surgical patients when adding the non-routinely collected variables needs to justify the extra effort/expense needed to collect those variables. At the same time, the calibration curve is used to visualize the results of the goodness of matching test. It can more intuitively display the degree of match between the clinical prediction situation and the actual situation.

This study has the following limitations: (1) The prediction model is a single-center trial, and the fit and discrimination of the prediction model need to be verified by a multi-center trial; (2) This research only studies general surgery, orthopedics, urology, patients undergoing elective surgery in hepatobiliary surgery, no detailed study of specific surgical methods has been carried out, and patients from other relevant surgical departments are to be included in the future to complete the study; (3) We test 33 variables in univariate analyses and they enter 26 variables into the Multivariable logistic regression model, which maybe place the model at high risk for overfitting in this relatively small dataset, therefore, it need to be required further verification; (4) The limitations of geographical and environmental factors. (5) Uneven fitting of the validation curves, which can be caused by too small a sample size in the validation set. We will show the stable efficacy of the predictive model by increasing the sample size. (6) The sample size of the validation group is small (199 cases), which may affect the external validation effect of the model. (7) The study follows patients for POD occurrence within 1 to 7 days post-surgery. While this captures immediate postoperative delirium, it does not account for late-onset POD, which can occur beyond the first week after surgery. A longer follow-up period would provide a more comprehensive assessment of POD risk.

## Conclusion

This clinical prediction model constructed by screening independent risk factors for POD has a good predictive performance, which can guide the screening of high-risk groups of POD patients in clinical work, and provide references for early intervention and treatment of POD.

## Data Availability

The original contributions presented in the study are included in the article/supplementary material. Further inquiries can be directed to the corresponding authors.
